# Prospective evaluation of the PRIMARY score using [^18^F]PSMA-1007 PET/MRI in second-line prostate biopsies after negative MRI

**DOI:** 10.1007/s00259-026-08070-3

**Published:** 2026-07-29

**Authors:** Mads Ryø Jochumsen, Lise Medrud, André H. Dias, Mette Abildgaard Pedersen, Christina Stilling, Kirsten Bouchelouche, Peter Iversen, Joan Fledelius, Lars Poulsen Tolbod, Louise Forsmann Grønnemark, Stefan Vind-Kezunovic, Morten Heebøll Andersen, Kasper Drimer Berg

**Affiliations:** 1 Department of Nuclear Medicine, Aarhus University Hospital, Palle Juul-Jensens Boulevard 165, Aarhus, 8200 Aarhus N Denmark; 2 Department of Clinical Medicine, Aarhus University, Aarhus, Denmark; 3 Department of Radiology, Aarhus University Hospital, Aarhus, Denmark; 4 Department of Pathology, Aarhus University Hospital, Aarhus, Denmark; 5 Department of Urology, Aarhus University Hospital, Aarhus, Denmark; 6 Department of Urology, Gødstrup Hospital, Herning, Denmark

**Keywords:** PSMA PET/MRI, Prostate cancer, Biopsy guidance, PRIMARY score, [^18^F]PSMA-1007

## Abstract

**Purpose:**

PSMA PET has been proposed for guiding targeted prostate biopsies. As the rate of clinically significant prostate cancer (csPCa) is positively correlated with PSA density (PSAd), a potential application for PSMA-guided biopsies may be men with negative MRI-first (PI-RADS-1-2) but increased PSAd. The PRIMARY scoring system, using the intraprostatic PSMA-pattern was developed and tested using [^68^Ga]Ga-PSMA-11 and proposed to be universal across PSMA-ligands, however no data on [^18^F]PSMA-1007 for biopsy guidance exists. Thus, the aim of this study was to evaluate the PRIMARY score for prostate biopsy guidance using [^18^F]PSMA-1007 in a prospective clinical setup.

**Methods:**

91 patients were prospectively enrolled for [^18^F]PSMA-1007 PET/MRI, 86 of these for biopsy guidance after negative MRI or non-csPCa biopsies from PI-RADS 3–5 lesions and persistent suspicion of csPCa. Transperineal MRI/ultrasound fusion target biopsies were performed predominantly from PRIMARY 3–5 lesions. Nine patients with PRIMARY 1–2 did not undergo target biopsies. CsPCa was defined as ISUP grade ≥ 2.

**Results:**

In a per patient analysis, 23.3% (20/86) were diagnosed with csPCa. In line with previous studies, the rate of csPCa increased with higher PRIMARY score in a per lesion analysis, constituting 0% (0/1), 0% (0/8), 7.2% (7/97), 60% (15/25), and 60% (6/10) for PRIMARY 1, 2, 3, 4, and 5 lesions, respectively. The 7.2% PRIMARY-3 lesions containing csPCa exhibited common characteristics such as anterior location or lack of well-defined separation from the peripheral zone.

**Conclusion:**

We evaluated the PRIMARY score for use with [¹⁸F]PSMA-1007 and identified benign features and potential “red flags” for PRIMARY-3 lesions. We found csPCa in 23.3% of patients with negative MRI or non-csPCa biopsies from PI-RADS 3–5 lesions and persistent suspicion of csPCa.

**Supplementary Information:**

The online version contains supplementary material available at https://doi.org/10.1007/s00259-026-08070-3.

## Introduction

The current standard-of-care work-up for patients under suspicion of prostate cancer (PCa) is magnetic resonance imaging (MRI) using PI-RADS score [1]. An MRI-first approach, where only PI-RADS-3-5 lesions are biopsied, reduce the number of patients getting unnecessary biopsies, detect more clinically significant PCa (csPCa), defined as International Society of Urological Pathology (ISUP) grade ≥ 2 [2], and reduce detection of non-csPCa (ISUP Grade 1) compared to systematic biopsies [3–6]. The PI-RADS grading system has a high sensitivity of 93–96% for detecting csPCa when PI-RADS-3-5 lesions are biopsied [7]. However, this implies a 4–7% risk of underdiagnosing csPCa, and hence a group of patients with a normal MRI, might be undertreated [7].

A recent review found the compiled risk of csPCa for patients with PI-RADS-1-2 lesions dependent on the prostate-specific antigen (PSA) density (PSAd) [8]. Patients with low (< 0.10 ng/mL/cm³), intermediate (0.10–0.20 ng/mL/cm³), and high (≥ 0.20 ng/mL/cm³) PSAd had csPCa risks of 4%, 11%, and 24%, prompting biopsy recommendations of “no biopsy,” “consider no biopsy,” and “highly consider systematic selected-site biopsies,” respectively [8]. An alternative strategy might be second-line targeted biopsies, guided by prostate-specific membrane antigen (PSMA) positron emission tomography (PET). This strategy in second-line might be preferable compared to systematic biopsies to decrease the number of biopsies, potentially increase detection of csPCa, and furthermore reduce the detection of non-csPCa. However, only few studies have used PSMA PET for detection of PCa [9–14] and the clinical role of PSMA PET/MRI is continuously discussed [15–17]. A five-point grading system (PRIMARY score) using the intraprostatic PSMA-pattern was developed by Emmett et al. [12] and further validated for biopsy guidance across different PI-RADS scores using [^68^Ga]Ga-PSMA-11 [13, 14, 18] but only retrospectively evaluated using [^18^F]PSMA-1007 [19]. Depending on patient selection, csPCa was found in 0–9% of PRIMARY-1 lesions, 0–27% of PRIMARY-2 lesions, 6–38% of PRIMARY-3 lesions, 39–76% of PRIMARY-4 lesions and 100% of PRIMARY-5 lesions [12, 14]. Recently, results from the PRIMARY-2 trial were published, randomizing patients with equivocal or non-suspicious findings on MRI to either systematic prostate biopsies or [^68^Ga]Ga-PSMA-11 PET/CT guided biopsies. PSMA-guided biopsies safely halved the number of men requiring biopsy, significantly reduced the non-csPCa detection without compromising the diagnosis of csPCa [20, 21].

[^18^F]PSMA-1007 is a widely used PSMA-tracer with potential advantages compared to [^68^Ga]Ga-PSMA-11 for local prostate cancer detection due to its low urinary bladder activity and the shorter positron range compared to ^68^Ga-labelled tracers, leading to less partial-volume effects and hence higher resolution and sharper PET-images [22]. Besides, ^18^F-labelled tracers enable large batch cyclotron production, and the longer half-life of the isotope allows long range tracer distribution.

The PRIMARY score has been proposed to be universal across PSMA-ligands, but solid data for [^18^F]PSMA-1007 is lacking. This study aimed to evaluate the PRIMARY score for prostate biopsy guidance using [^18^F]PSMA-1007 in a prospective clinical setting.

## Materials and methods

### Patient population

**Fig. 1:**
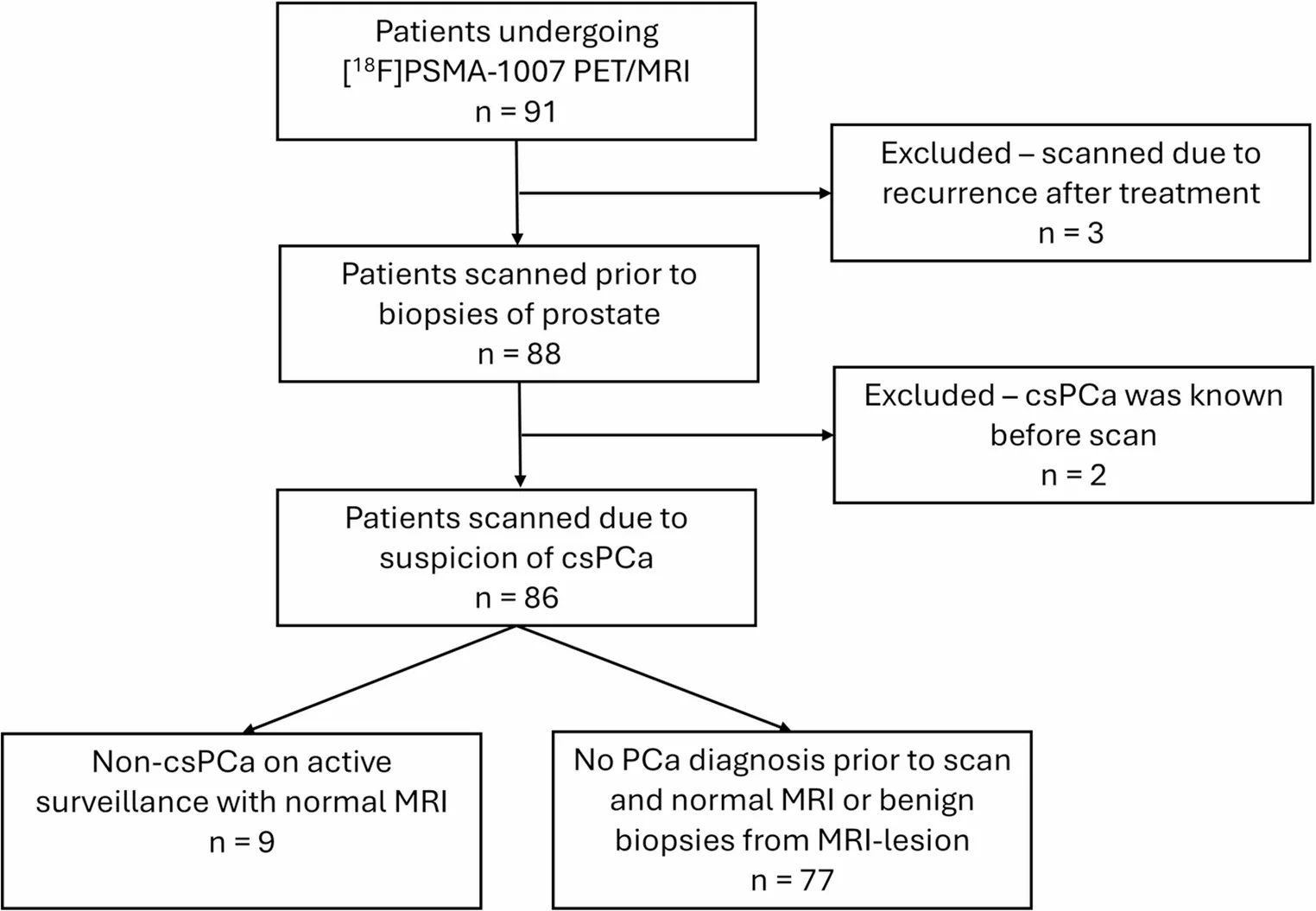
Flow chart for definition of the cohort of included patients

We prospectively enrolled all men (*n* = 91) undergoing [^18^F]PSMA-1007 PET/MRI from December 2023 to December 2025. Inclusion criteria in the present study was [^18^F]PSMA-1007 PET/MRI performed due to 1: men undergoing evaluation for prostate cancer with a negative MRI (PI-RADS 1–2) with persistent suspicion of csPCa due to an elevated PSAd; 2: men with benign biopsies or non-csPCa from PI-RADS 3–5 lesions with persistent suspicion of overlooked csPCa due to an elevated PSAd; and 3: men with non-csPCa followed in an active surveillance protocol, where the MRI scan prior to re-biopsies were negative (PI-RADS 1–2). Three patients were excluded as they were scanned for detection of biochemical recurrence after primary treatment and not for biopsy guidance (Fig. 1). Furthermore, two patients with known csPCa (ISUP 2) were excluded in the present study. Thus, the present study included 86 patients who underwent [^18^F]PSMA-1007 PET/MRI for biopsy-guidance. Of these, 9 patients (10.5%) were under Active Surveillance for ISUP 1 low-risk PCa without suspicion on follow-up MRI. The PSMA PET/MRI was then performed before re-biopsy of the prostate.

Eighty-five patients were included in a study and biobank, approved by the institutional review board (Central Denmark Region Committees on Health Research Ethics (1-10-72-262-21)), and signed a written informed consent. Six patients were not included in the abovementioned study, but retrospective study of these patients was approved by the local institutional board at Aarhus University Hospital.

### Imaging

Patients fasted three hours before the scan. Scans were performed on a 3 Tesla GE SIGNA PET/MRI scanner (GE Healthcare, Waukesha, Wisconsin, USA). 2.5 MBq/kg [^18^F]PSMA-1007 was injected. Intramuscular glucagon injection was administered immediately before scan start for reduction of bowel peristalsis and hence motion artifacts of the prostate MRI. Using a 3-plane localizer sequence for planning, the PET scan was centred on the prostate. Initially, a T2 weighted axial sequence was performed for maximal glucagon effect. A 20-minute PET acquisition from 60 min (± 10 min) post-injection was used for reconstruction of SUV images.

Images were reconstructed using VuePointFX SharpIR (using ordered subset expectation maximum reconstruction (OSEM) algorithm with time-of-flight (ToF) and point-spread-function (PSF), 3 mm 2D Gaussian and [1 2 1] axial convolution post-filters) with 1.6 × 1.6 × 2.8 mm^3^ voxels. An MRI-derived AC-Map was used for attenuation correction.

Bi-parametric MRI with coronal and sagittal T2 and diffusion weighted sequences were performed simultaneously with the 20-minute PET acquisition. Later, we added a late T2 weighted axial sequence within the last minutes of the PET acquisition due to experiencing a few cases with misalignment between PET and the early T2 weighted axial sequence caused by patient-movement or alterations in bladder and rectal volumes.

### Image analysis

[^18^F]PSMA-1007 PET/MRI scans were analyzed in daily clinical routine on the PSMA PET dual-reading track with MRI-radiologist (LM) and a nuclear medicine specialist (MRJ, AHD, KB, JF or PI). PET scan analysis was performed using HERMIA Hybrid Viewer PDR 6.1.4 (Hermes Medical Solutions, Stockholm, Sweden). The PRIMARY scoring system from [^68^Ga]Ga-PSMA-11 was adapted 1:1, as there was no data on [^18^F]PSMA-1007 at that point. Guided by the nuclear medicine specialist, the expert radiologist (LM) performed delineation of PRIMARY-3-5 lesions on the MRI scan using MIM Software Version 7.2.8 (MIM Software Inc., Cleveland, Ohio, USA).

MRJ retrospectively performed second read of all scans and reclassification of PRIMARY-lesions where performed only in obvious cases in consensus between MRJ, LM and AHD. The final PRIMARY score was changed in five cases; in four cases because second read of the scan revealed that the lesion was PRIMARY-2, and not PRIMARY-3 as the uptake was definitely less than twice TZ background, and in one case from PRIMARY-4 to PRIMARY-5 as the SUVmax was objectively above 12.

### Biopsies

Transperineal MRI/ultrasound fusion target biopsies were performed by trained urologists (in most cases SVK or MHA), using BK-5000 ultrasound system with a BK Medical E14CL4b transducer (BK-medical, GE Healthcare, Waukesha, Wisconsin, USA).

Patients were positioned in lithotomy position on the examination table. The [^18^F]PSMA-1007 PET/MR image was then aligned with the transrectal ultrasound image using the MIM-software Symphony Freehand TPBx version 7.2 (MIM Software Inc., Cleveland, Ohio, USA) and 3D guidance trakSTAR fusion equipment (Ascension Technology Corporation, Burlington, Vermont, USA). Typically, 3–4 biopsy cores were collected transperineally from each suspicious PRIMARY-lesion (generally PRIMARY-3-5 lesions).

### Histopathological parameters and immunohistochemistry

Prostate biopsies were fixed in formalin and paraffin embedded. Three-micron sections were cut, and slides were stained with Hematoxylin and eosin using VENTANA HE 600 Systems using routine diagnostic protocol. Four PRIMARY 5 lesions with high PSMA-uptake and two PRIMARY 4 lesions with benign histopathology were selected for PSMA-immunohistochemistry (IHC), conducted on Benchmark Ultra plus (Ventana, Roche tissue diagnostics) and visualized with OptiView DAB kit according to the manufacturer’s standard settings and reagents. Heat induced epitope retrieval was conducted using a high pH buffer for 48 min. The primary antibody PSMA (clone 3E6, mouse, Agilent M3620) was used in a 1:50 dilution and incubated for 32 min.

Biopsies were retrospectively re-evaluated and scored regarding inflammation. The inflammation score evaluated inflammatory infiltration and aggressiveness as described by Irani et al. [23]. In short, inflammatory infiltration was graded as 0 if no inflammatory cells were present, 1 in case of scattered inflammatory cell infiltration within the stroma without lymphoid nodules, 2 as non-confluent lymphoid nodules, and 3 as large inflammatory areas with confluence of infiltrates. Inflammatory aggressiveness was graded as 0 if there were no contact between inflammatory cells and glandular epithelium, 1 in case of contact between the inflammatory cell infiltrate and glandular epithelium, 2 interstitial inflammatory infiltration associated with a clear but limited glandular epithelium disruption (< 25%), and 3 was defined as glandular epithelium disruption on more than 25% of the examined material. Furthermore, signs of acute inflammation, defined as the presence of neutrophilic granulocytes, as well as eosinophilic inflammation were recorded.

PSMA positivity was visually evaluated according to Vetrone et al. [24] and a score between 0 and 3 + was given. 0 was defined as negative, 1 + as weak, 2 + as moderate, and 3 + as strong positivity.

### Statistical analysis

In general, descriptive statistics were applied. Before parametric analysis, data was tested for normality using QQ-plots and histograms. Students t-test of SUVmax for csPCa vs. non-csPCa and benign findings combined was performed on log-transformed data due to non-normal distribution. Receiver operating characteristic (ROC) analyses were used for calculating the area under the curve (AUC). P values < 0.05 were considered statistically significant. Statistical analysis was performed using StataNow version 19.5.

## Results

Ninety-one patients were enrolled and examined with [^18^F]PSMA-1007 PET/MRI. Three patients were excluded from analysis as they were scanned due to biochemical recurrence after primary treatment with radical prostatectomy, cryotherapy and Nanoknife electroporation ablation [25], respectively. Two patients with known csPCa (ISUP 2) were also excluded. The remaining eighty-six patients with negative MRI-first (PI-RADS-1-2) or previous biopsies from PIRADS 3–5 lesions without csPCa, but persistent suspicion of csPCa were scanned for biopsy guidance and constitutes the cohort of the present study (Fig. 1). Baseline characteristics of these eighty-six patients are found in Table 1.

**Table 1:** Baseline characteristics of patients

Age (years)	
Median (range)	66 (48–79)
PSA density (ng/mL/cm³)	100% (86/86)
Median (range)	0.25 (0.06–0.6)
<0.10	3.5% (3/86)
0.10–0.15	2.3% (2/86)
0.15–0.20	12.8% (11/86)
>0.20	81.4% (70/86)
Pre-scan PSA (ng/mL)	100% (86/86)
Median (range)	16.0 (3.0-68.5)
<10	22.1% (19/86)
10–20	45.3% (39/86)
>20	32.6% (28/86)
Previous MRI	100% (86/86)
PI-RADS-1-2	89.5% (77/86)
PI-RADS-3	1.2% (1/86)
PI-RADS-4	5.8% (5/86)
PI-RADS-5	3.5% (3/86)
Previous biopsies	65.1% (56/86)
Benign	83.9% (47/56)
ISUP Grade 1	16.1% (9/56)

In a per patient analysis, 23.3% (20/86) were diagnosed with csPCa.

For patients with PSAd below 0.10 ng/mL/cm³ and between 0.10 and 0.15 ng/mL/cm³ we found csPCa in 0% (0/3) and (0/2), respectively. For patients with PSAd between 0.15 and 0.20 ng/mL/cm³, we found csPCa in 18.2% (2/11), while we found csPCa in 25.7% (18/70) of patients with PSAd ≥ 0.20 ng/mL/cm³. All patients with csPCa had PSAd ≥ 0.17 ng/mL/cm³.

Nine patients did not undergo targeted biopsies as only PRIMARY-1-2 pattern was present.

Four patients with benign [^18^F]PSMA-1007 PET/MRI-guided biopsies underwent a robot-assisted simple prostatectomy a.m. Freyer due to lower urinary tract symptoms. In all four cases the histopathological evaluation was with benign findings (Table 2).

**Table 2:** Data on final histopathology on a patient level for[¹⁸F]PSMA-1007-guided biopsies and radical prostatectomy, respectively

Highest PRIMARY score		PRIMARY 1–2	PRIMARY 3	PRIMARY 4–5
PSMA-guided Biopsies	89.5% * (77/86)	15.1% (13/86)	57.0% (49/86)	27.9% (24/86)
Benign	61.0% (47/77)	6.4% (3/47)	85.1% (40/47)	8.5% (4/47)
ISUP Grade 1	11.7% (9/77)	11.1% (1/9)	55.6% (5/9)	33.3% (3/9)
ISUP Grade 2	15.6% (12/77)	0.0% (0/12)	16.7% (2/12)	83.3% (10/12)
ISUP Grade 3	3.9% (3/77)	0.0% (0/3)	66.7% (2/3)	33.3% (1/3)
ISUP Grade 4	6.5% (5/77)	0.0% (0/5)	0.0% (0/5)	100% (5/5)
ISUP Grade 5	1.3% (1/77)	0.0% (0/1)	0.0% (0/1)	100% (1/1)
Prostatectomy specimens **	17.4% (15/86)	0.0% (0/15)	40% (6/15)	60% (9/15)
Benign	26.7% ** (4/15)	0.0% (0/4)	100% (4/4)	0.0% (0/4)
ISUP Grade 1	0.0% (0/15)	-	-	-
ISUP Grade 2	26.7% (4/15)	0.0% (0/4)	0.0% (0/4)	100% (4/4)
ISUP Grade 3	33.3% (5/15)	0.0% (0/5)	40% (2/5)	60% (3/5)
ISUP Grade 4	6.7% (1/15)	0.0% (0/1)	0.0% (0/1)	100% (1/1)
ISUP Grade 5	6.7% (1/15)	0.0% (0/1)	0.0% (0/1)	100% (1/1)

*Nine patients with PRIMARY 1–2 did not undergo target biopsies

**Four patients with benign [^18^F]PSMA-1007 PET/MRI-guided biopsies underwent a robot-assisted simple prostatectomy a.m. Freyer due to lower urinary tract symptoms. Eleven patients underwent radical prostatectomy

### Prospective evaluation of PRIMARY score using [^18^F]PSMA-1007

**Fig. 2:**
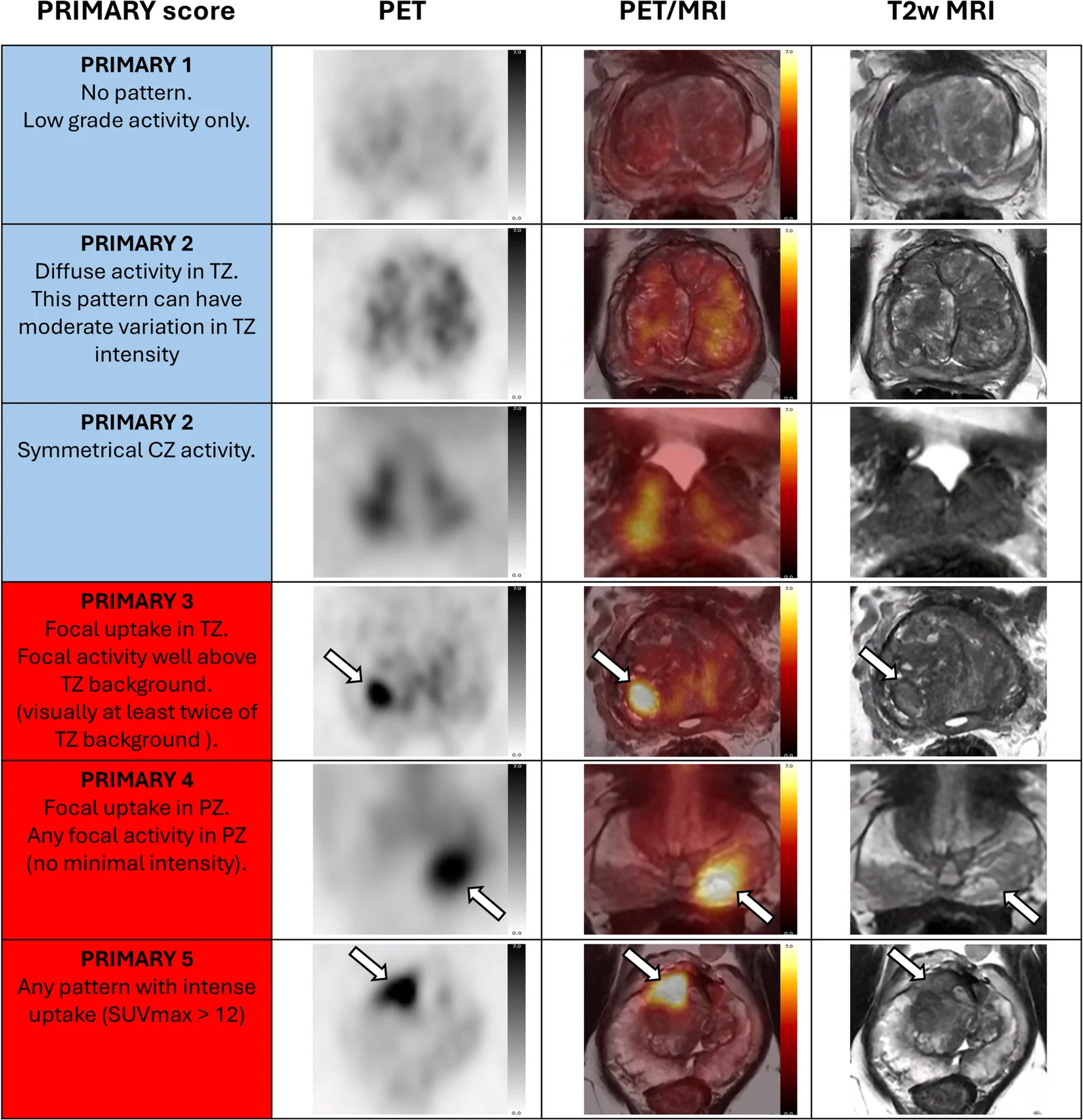
[^18^F]PSMA-1007 PET/MRI examples for each PRIMARY score. SUV-scale range [0.0–7.0]. Transitional zone (TZ), central zone (CZ), peripheral zone (PZ)

Figure 2 shows examples of the intraprostatic [^18^F]PSMA-1007 pattern for each PRIMARY score.

In total, 150 PRIMARY-lesions were found in the present study; 18 PRIMARY-1-2 lesions, 97 PRIMARY-3 lesions, 25 PRIMARY-4 lesions and 10 PRIMARY-5 lesions. In a per lesion analysis, 141 (94.0%) were biopsied including nine PRIMARY-1-2 lesions and all PRIMARY-3-5 lesions. CsPCa was found in 19.9% (28/141) of biopsied lesions, ISUP Grade 1 in 9.9% (14/141), while 70.2% (99/141) were benign. Of the nine patients who underwent target biopsy from PRIMARY-1-2 lesions, five cases were due to suspicion on MRI in that location and in four cases because second read of the scan revealed that the lesion was PRIMARY-2, and not 3 as the uptake was less than twice TZ background. Biopsied PRIMARY 1–2 lesions were benign in eight cases whereas one had non-csPCa.

**Fig. 3:**
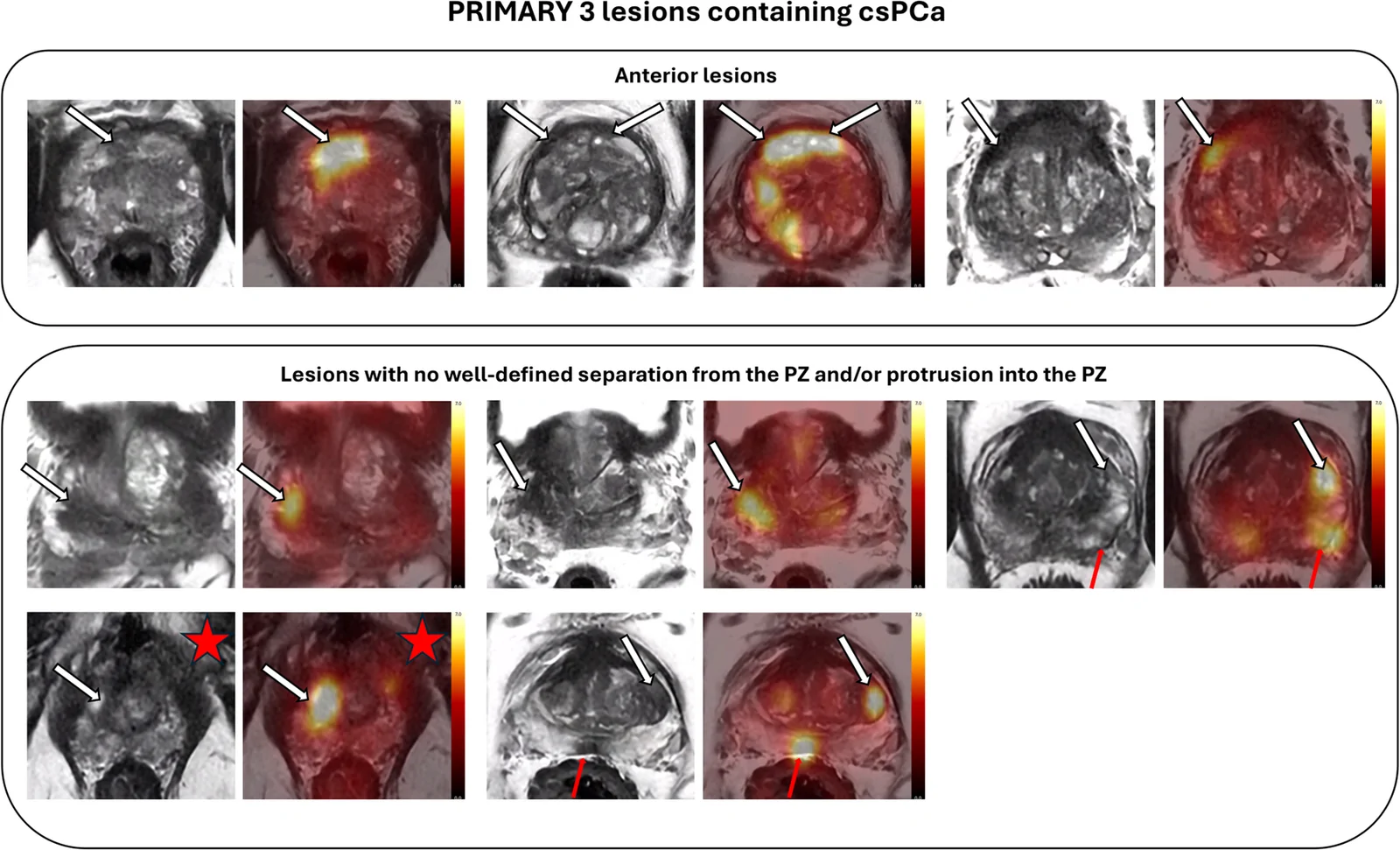
All cases of PRIMARY-3 lesions with csPCa (marked with broad white arrows). The case in the lower left row (marked with red star) is an excluded patient with previously known ISUP 2, also in a PRIMARY 3 lesion. SUV-scale range [0.0–7.0]. PRIMARY-4 lesions are visible in the same axial slice in two patients (marked with thin red arrows)

For biopsied PRIMARY-3 lesions, 84.5% (82/97) were benign and 8.3% (8/97) were ISUP Grade 1. These lesions usually correspond to well-defined nodules within the transitional zone (TZ) (Fig. 2, PRIMARY-3 example). The seven cases (7.2%) of PRIMARY-3 lesions containing csPCa are shown in Fig. 3 along with an excluded patient with previously known ISUP 2, also in a PRIMARY 3 lesion. Certain common characteristics of these were identified; often not well defined or with irregular shape and they are all located in the periphery of the TZ, distributed as three anterior lesions and five lesions with ill-defined separation from the peripheral zone (PZ) or protrusion into the PZ (Fig. 3).

**Fig. 4:**
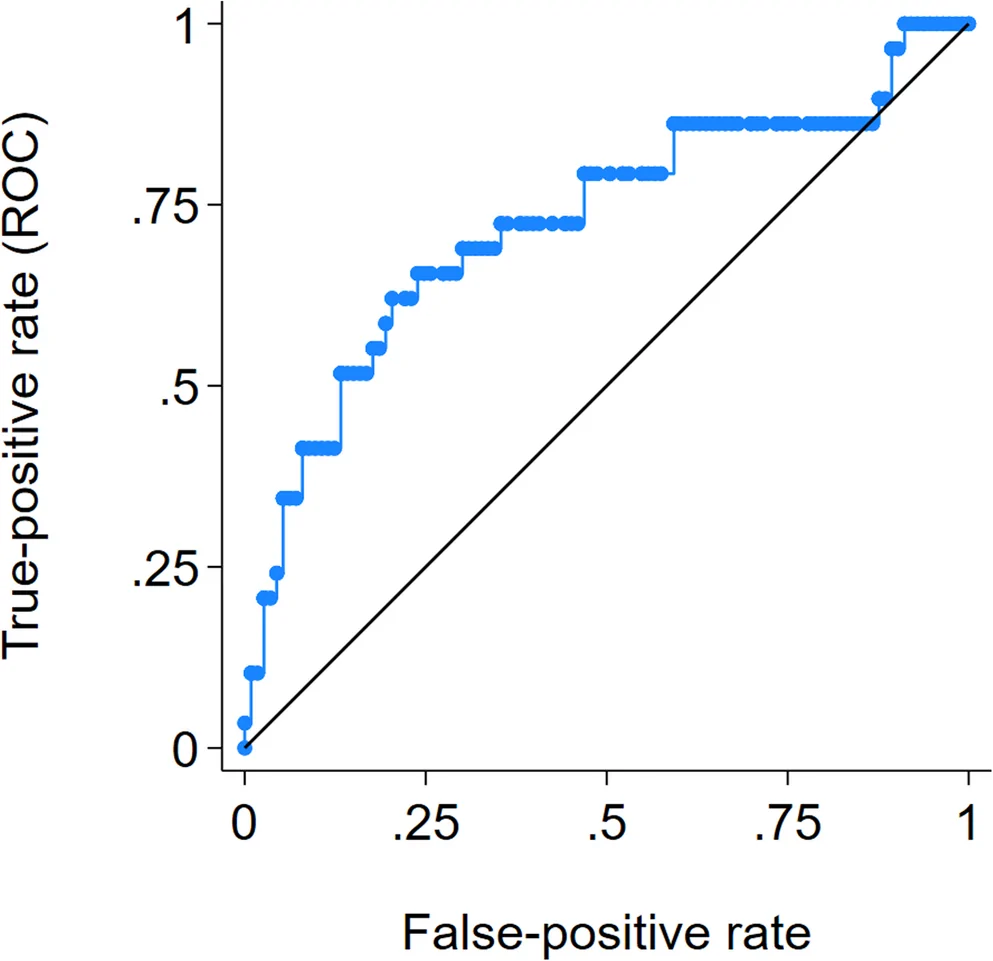
ROC curve for [^18^F]PSMA-1007 SUVmax for the ability to detect csPCa (AUC = 0.72)

SUVmax was significantly higher for lesions with csPCa (median 8.74, interquartile range (IQR) [6.88–11.32]) than for non-csPCa and benign lesions combined (median 6.80, IQR [6.11–7.97]) (*p* < 0.0001). The seven PRIMARY-3 lesions, containing csPCa, had a median SUVmax of 7.80, range [6.52–10.72]. ROC analysis of SUVmax to detect csPCa is shown in Fig. 4 (AUC = 0.72).

Table 3 shows the distribution of PRIMARY-lesions across examined patients in addition to the percentage of csPCa for each PRIMARY score, compared to previous studies on PRIMARY score. Final histopathology on a patient level for PSMA-guided biopsies and radical prostatectomy is provided in Table 2. Of the patients who underwent radical prostatectomy, there was complete agreement between target biopsy- and radical prostatectomy ISUP grade group in 86.7% (13/15). The last 2 patients were scored as ISUP 4 on target biopsies and downgraded to ISUP 3 on radical prostatectomy histopathology, and hence still presenting csPCa.

**Table 3:** Number of lesions biopsied and rate of csPCa for each PRIMARY score. Results from the present study compared to the two previous studies using PRIMARY score for biopsy guidance. Although 150 lesions were PRIMARY scored, only 141 lesions (94.0%) were biopsied explaining the discrepancy between total number of PRIMARY-lesions and number of lesions in Table 3

	Jochumsen et al. (present study)		Emmett et al.	Shi et al.
	96.0% PI-RADS-1-2		PI-RADS-2-5	PI-RADS-3
	Lesions	csPCa (%)	csPCa (%)	csPCa (%)
PRIMARY-1	1	0	8.5	0
PRIMARY-2	8	0	27	0
PRIMARY-3	97	7.2	38	6.3
PRIMARY-4	25	60.0	76	38.5
PRIMARY-5	10	60.0*	100	100

*One lesion was regarded csPCa as it was later re-biopsied finding csPCa (the lesion had then “regressed” to PRIMARY-4 (SUVmax = 11.47))

Nine patients (10.5%) had PI-RADS-3-5 lesions on the MRI scan performed prior to inclusion. In contrast, 96.0% (144/150) of the PRIMARY-lesions on [^18^F]PSMA-1007 PET/MRI were scored as PI-RADS-1-2 illustrating that the PI-RADS and PRIMARY-lesion were not overlapping in most cases.

### PSMA-avid lesions with benign histopathology

Pathologists reported inflammation in 40.4% (40/99) of all biopsied PRIMARY-lesions with benign histopathology, indicating that inflammation can cause some of the benign PSMA-uptake. However, inflammation is not mandatorily reported in pathology reports, thus the percentage of false-positive PSMA-uptake caused by inflammation might be higher.

We found both PRIMARY-4 and noteworthy PRIMARY-5 lesions with benign pathology findings. As this contrasts with previous reported data, we performed PSMA-immunohistochemistry (IHC) on four PSMA-avid PRIMARY-5 lesions and two PRIMARY-4 lesions, all with benign histopathology, to explore the reason for high PSMA-uptake in benign tissue.

**Fig. 5:**
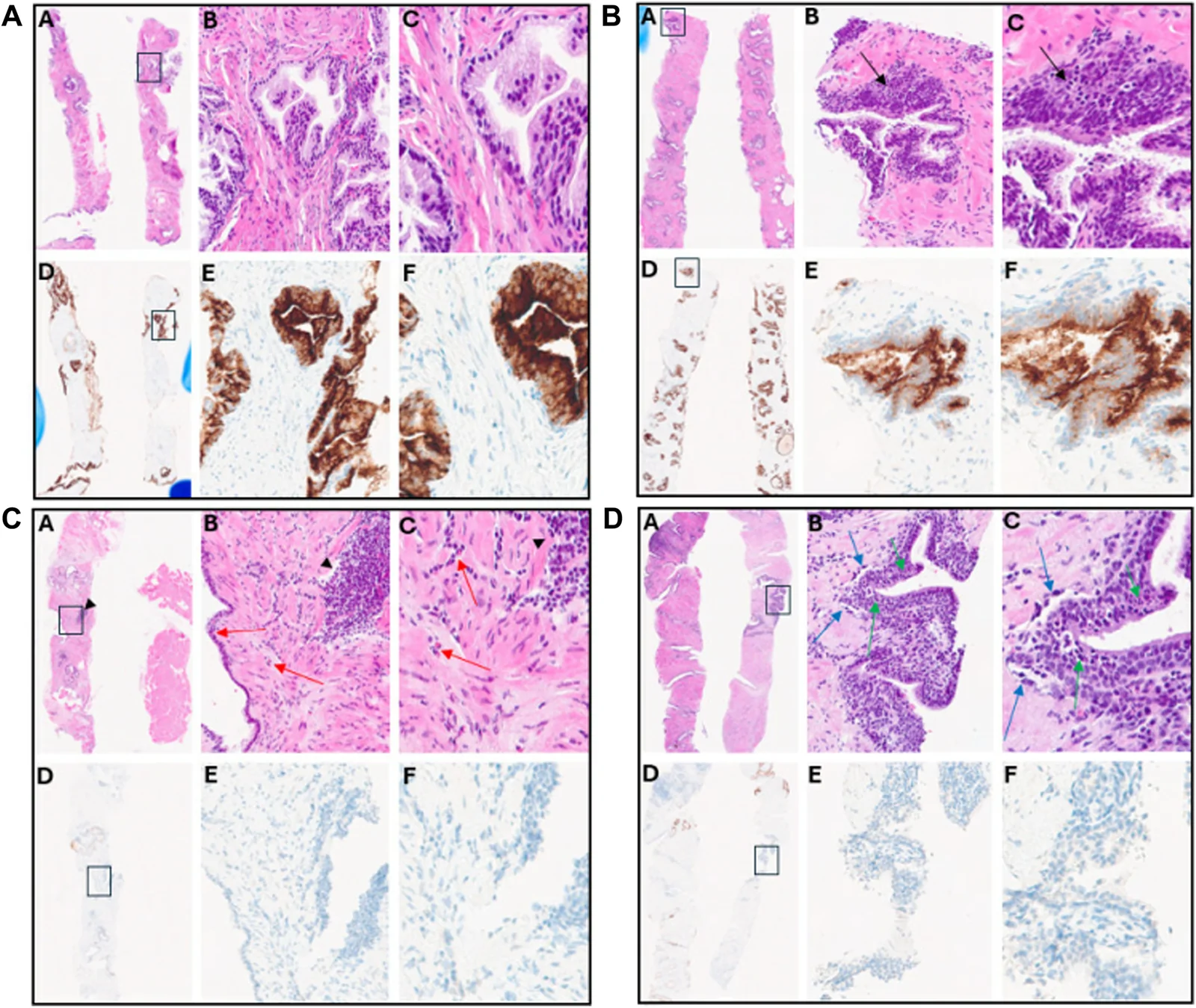
Prostate biopsies from four patients with PRIMARY-4 and 5 with benign histopathology. For each patient: Upper row: sections stained with hematoxylin and eosin, lower row: sections with PSMA-immunohistochemistry. A and D: section overview, B and E: magnification 10x, C and F: magnification 20x. **A **Patient 50: PRIMARY-5 lesion, SUVmax = 35, low inflammation (Inflammatory score 1 − 0; scattered inflammatory cells within the stroma and no contact between inflammatory cells and glandular epithelium) and very high PSMA-expression (PSMA-IHC positivity = 3 + in 85% of glandular epithelium). **B** Patient 8: PRIMARY-5 lesion, SUVmax = 12.53, relatively low inflammation (Inflammatory score 1–1; scattered inflammatory cells within the stroma with contact between inflammatory cells and glandular epithelium (black arrows)) and very high PSMA-expression (PSMA-IHC positivity = 3 + in 90% of glandular epithelium). **C** Patient 24: PRIMARY-4 lesion, SUVmax = 5.85, intermediate inflammation (Inflammatory score 2 − 1; non-confluent lymphoid nodules (black arrowheads) with contact between inflammatory cells and glandular epithelium). Eosinophil infiltration (red arrows). Low PSMA-expression (PSMA-IHC positivity = 2 + in only 1% of glandular epithelium). **D** Patient 15: PRIMARY-5 lesion, SUVmax = 12.18, high inflammation (Inflammatory score 2–2; non-confluent lymphoid nodules and interstitial inflammatory infiltrate associated with glandular epithelium disruption below 25% (blue arrows)). Neutrophil infiltration (green arrows). Low PSMA-expression (PSMA-IHC positivity = 3 + in less than 5% of glandular epithelium)

On re-analysis of the selected PRIMARY-4 and 5 lesions, varying degree of inflammation and inflammation type were reported in all lesions (Table 4; Fig. 5).

**Table 4:** Overview of the re-analysis of selected PRIMARY-4 and 5 lesions with benign histopathology. Inflammatory scores [23], inflammation types, as well as PSMA-IHC positivity [24] are provided. Transitional zone (TZ), peripheral zone (PZ), immunohistochemistry (IHC)

Patient	PRIMARY	SUVmax	Zone	Inflammatory score	Inflammation type	PSMA-IHC positivity
2	4	4.53	PZ	1–2	Acute and chronic	3 + in 30%
8	5	12.53	PZ	1–1	Acute and chronic	3 + in 90%
15	5	12.18	PZ	2–2	Acute and chronic	3 + in 2%
15	5	15.88	TZ	2 − 1	Chronic	3 + in 2%
24	4	5.85	PZ	2 − 1	Eosinophilic	2 + in 1%
50	5	35	TZ	1 − 0	Chronic	3 + in 85%

On PSMA-IHC, the PSMA-uptake was found only in benign prostatic epithelial cells (Fig. 5, D, E, and F), with no obvious association between degree or type of inflammation and the degree of PSMA-expression.

### [^18^F]PSMA-1007 PET- and MRI-negative clinically significant prostate cancer

**Fig. 6:**
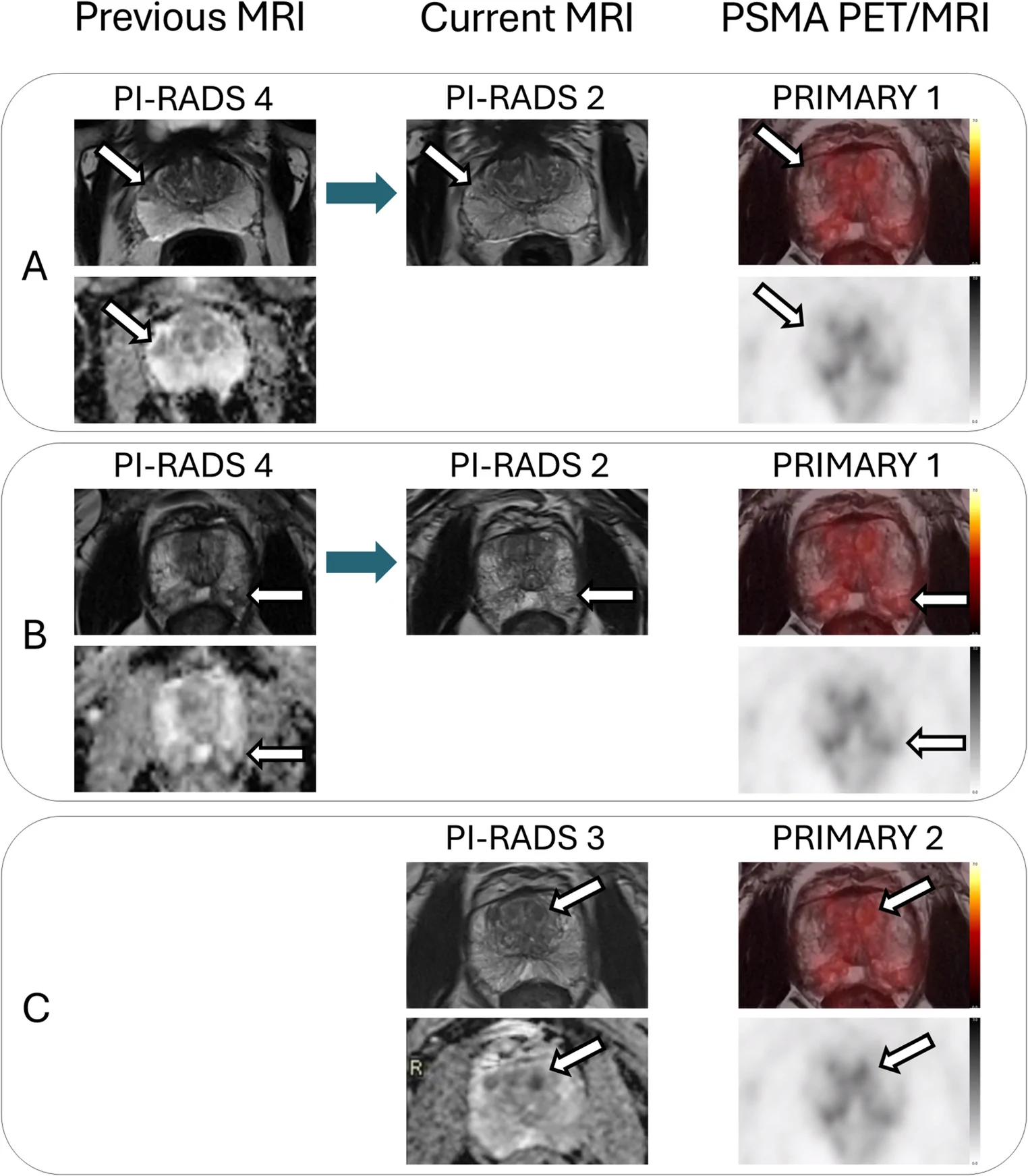
Axial MRI (T2 weighted sequence above and Apparent Diffusion Coefficient (ADC) map below) and PSMA PET/MRI images (transaxial PET/MRI fused images above and transaxial PET images below) of a patient with known ISUP 2 csPCa, currently both negative on MRI (PI-RADS-2) and [^18^F]PSMA-1007 PET (PRIMARY-1/2). SUV-scale range [0.0-7.0]

One patient needs to be highlighted even though he was excluded from the current study, due to previously known csPCa, as he was an exception for the detection rates presented in Table 3. On MRI-first, he was diagnosed with ISUP Grade 2 in a right PZ PI-RADS-4 lesion with less than 5% Gleason pattern 4 (Fig. 6, row A). Furthermore, a left PZ PI-RADS-4 lesion with benign biopsies (Fig. 6, row B).

He started active surveillance, and subsequent follow-up MRI showed regression of both previous PI-RADS-4 lesions to PI-RADS-2 (Fig. 6, row A and B). Meanwhile, a small PI-RADS-3 lesion in the left anterior TZ appeared (Fig. 6, row C). Due to atypical appearance, [^18^F]PSMA-1007 PET/MRI was performed, presenting only PRIMARY-1/2 pattern (Fig. 6, row A, B, and C).

One of three biopsies from the PI-RADS-3 lesion (Fig. 6, row C) and two of three biopsies from the PI-RADS-2 lesion (previously PI-RADS-4) in the left PZ showed ISUP Grade 1, however 2 mm of the last biopsy from the left PZ PI-RADS-2 lesion showed ISUP Grade 4 (4 + 4) (Fig. 6, row B).

**Fig. 7:**
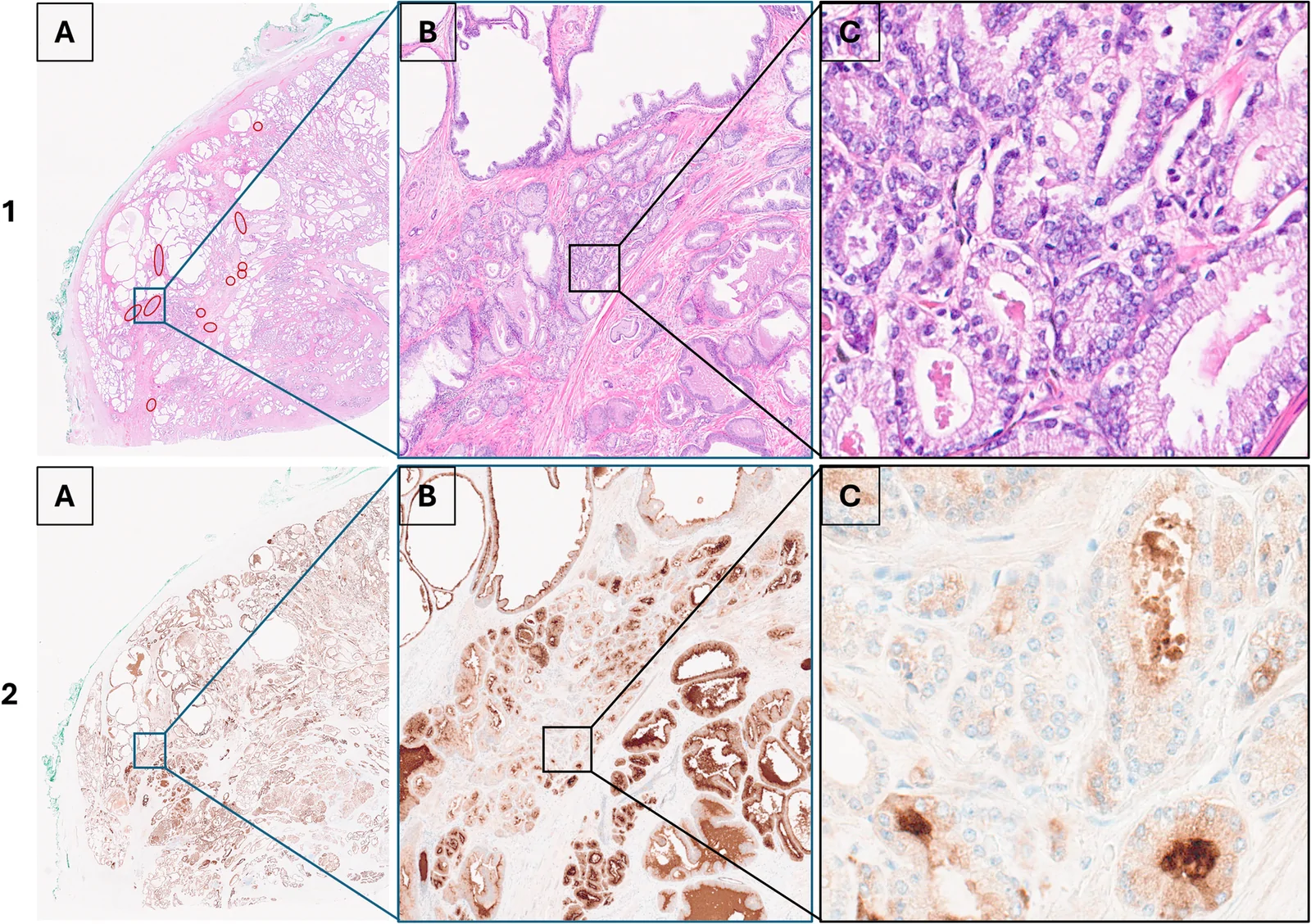
Prostate section from a patient with multiple microfocal tumor infiltrations (1 A, red circles). The tumor is currently negative on both MRI (PI-RADS-2) and [^18^F]PSMA-1007 PET (PRIMARY-1/2). Cancer cells had low PSMA-expression or PSMA-negativity (Row 2). Row 1: section stained with hematoxylin and eosin. Row 2: section with PSMA-immunohistochemistry. Columns: **A**: section overview, **B**: magnification 2.5x, **C**: magnification 20x

Radical prostatectomy pathology reported multiple microfocal ISUP Grade 2 (10% Gleason pattern 4) tumor infiltrations as illustrated in Fig. 7 (row 1), spread across approximately 25% of the prostate gland. PSMA-IHC of the radical prostatectomy revealed PSMA-negative disease (Fig. 7, row 2), a known phenomenon in at least 3.5% of PCa cases [26, 27] and also reported by Emmett et al. [12]. In this case, the tumor is both PSMA-negative (PRIMARY-1/2) and MRI-negative (PI-RADS-2).

## Discussion

### Prospective evaluation of PRIMARY score using [^18^F]PSMA-1007

In the present study, we prospectively evaluated the PRIMARY score using [^18^F]PSMA-1007 on PET/MRI. Important differences must be noted compared to previous reported data by Emmett et al. [12, 13] and Shi et al. [14], regarding the rate of csPCa for each PRIMARY score. First, the patient cohorts differ as the previous studies primarily assessed and characterized suspicious PI-RADS lesions. Emmett et al. studied PI-RADS-2-5 lesions, and Shi et al. studied PI-RADS-3 lesions, whereas we primarily included PI-RADS 1–2 lesions. Secondly, our focus was detection of lesions and biopsy guidance rather than characterization of known lesions. In the present study, most patients were examined because csPCa was suspected, typically due to increased PSAd, although a previous MRI and/or previous biopsies were benign or with non-csPCa. Thus, 96% of PRIMARY-lesions biopsied were PI-RADS-1-2 on MRI. This difference in a priori risk of csPCa explains some of the differences in the positive rate for each PRIMARY score between the studies. The overall increasing rate of csPCa with increasing PRIMARY score are, however, in line with previous studies, especially close to the data from PI-RADS 3 lesions from Shi et al. [14]. This underlines the clinical applicability of the PRIMARY score across different PSMA-tracers.

In our experience, separating PRIMARY-2 from PRIMARY-3 is the most challenging part of the PRIMARY score. Obvious cases of focal PSMA-avid areas well above TZ-background are easy to score as PRIMARY-3, and cases with homogenous TZ PSMA-uptake are quite easy to score as PRIMARY-2. However, many patients end up in a grey-zone with a very inhomogeneous PSMA-uptake in the TZ and still one or more focal well-defined PSMA-avid areas. In these cases, defining the TZ-background is challenging and observer-dependent, and since PRIMARY-3 is defined as twice the uptake of this background, the choice of reference area is critical. In the current setting of detection/biopsy guidance, we were cautious not to miss significant lesions and therefore defined the TZ-background as a TZ area of relatively homogenous low PSMA-uptake, occasionally upgrading lesions to PRIMARY-3 rather than downgrading to PRIMARY-2. A consensus about TZ-background definition is warranted.

In the present study, well-defined nodules (looking like benign prostatic hyperplasia (BPH)) well inside the TZ, turned out not to represent csPCa, despite PSMA-uptake well above TZ-background (Fig. 2, PRIMARY-3 example). Therefore, it might be possible to downgrade well-defined nodules, displaying typical BPH changes, well inside the TZ on future revisions of the PRIMARY score. PSMA-uptake in benign hyperplasia is a well-known pitfall, explaining why most biopsies from TZ foci are benign [28].

However, 7.2% of PRIMARY-3 lesions did contain csPCa, indicating that these lesions should not be disregarded (Fig. 3). Generally, these lesions were located superficially in the TZ, with both anterior lesions and lesions with no well-defined separation from the PZ or even protrusion into the PZ – these characteristics may be seen as “red flags” for PRIMARY-3 lesions. However, four PRIMARY-3 lesions close to the PZ/protrusion turned out to be benign, hence not all PRIMARY-3 lesions with these characteristics are malignant. In the present study, we used PET/MRI for optimal alignment between PET and prostate anatomy on MRI, which gives additional knowledge for the assessment as mentioned above, but also challenges using the exact same criteria for PRIMARY scoring. Some PRIMARY-3 lesions in Fig. 3 containing csPCa might have been scored as PRIMARY-4 on PSMA PET/CT due to location near the peripheral margin of the prostate gland, however PET/MRI fusion reveals that the lesion is inside the TZ or TZ-protrusion.

As reported by Emmett et al. [13] the inter-reader variability is considerable, and this is further amplified when such “red flags” are considered. PRIMARY scoring for biopsy guidance involves more than simply identifying the most avid lesion and should therefore be regarded as a specialized task requiring trained readers.

As PRIMARY-5 lesions are defined as SUVmax > 12, the SUVmax is an integral part of the score. Previous studies reported a 100% cancer detection rate for PRIMARY-5, whereas only 60% of PRIMARY-5 lesions in the present study were csPCa, indicating that SUVmax cut-off > 12 may be less predictive than reported in previous studies [12, 14]. The false-positive PRIMARY-4 and 5 lesions were of adequate size, so we expect all targets to be accurately sampled and PSMA-IHC of these biopsies revealed varying degrees of inflammation and PSMA-uptake located in benign prostatic epithelial cells (Fig. 5). Due to the shorter positron range of ^18^F compared to ^68^Ga, leading to less partial-volume effects, the optimal SUVmax cut-off for [^18^F]PSMA-1007 might be higher than for [^68^Ga]Ga-PSMA-11. However, the benign lesion with highest uptake in the present study had SUVmax = 25.93 and hence, no clear SUVmax threshold for defining a high-risk group could be established in our cohort. Different patient selection criteria do not seem to fully explain this deviation from previous reported data, and potentially, this clinically relevant differentiation between PRIMARY-4 and PRIMARY-5 on [^68^Ga]Ga-PSMA-11 is simply not transferable to [^18^F]PSMA-1007. However, the present cohort and especially the number of PRIMARY-5 lesions are too small for conclusions, and a larger patient cohort is needed to determine whether a clinically meaningful SUVmax cut-off can be determined for PRIMARY 5 using [^18^F]PSMA-1007. While this difference may reflect variations in PSMA-tracers or patient cohorts, it highlights that SUVmax alone is insufficient to reliably identify csPCa, and additional factors should be considered when interpreting PSMA PET for biopsy guidance.

In the context of the present study, where the goal is to potentially omit unnecessary biopsies in some patients, the key question is: “to biopsy or not to biopsy”. The proportion of csPCa relative to the combined non-csPCa and benign findings remains stable across all SUVmax cut-offs above 8.6. Below SUVmax = 8.6, the number of non-csPCa and benign biopsies increasingly exceeds the number of csPCa. This is supported by a ROC analysis showing a relatively equal rate of correctly classified patients (78-82.3%) for SUVmax cut-offs above 8.6 (highest at SUVmax = 10.72). However, with an AUC of 0.72, SUVmax cannot stand alone (Fig. 4). Hence, our data suggests that biopsy should be strongly considered for focal lesions with SUVmax > 8.6. Although the sensitivity and specificity in the present study are somewhat lower due to patient selection, this SUVmax cut-off is in line with the quantitative PRIMARY score (Q-PRIMARY), suggested by Gao et al., suggesting an SUVmax cut-off for detection of csPCa ranging from 7.09 to 7.91 depending on focal vs. diffuse uptake and TZ vs. PZ [19]. SUVmax seems to provide prognostic information and correlate with aggressiveness and a conjunction of SUVmax and PRIMARY score as proposed by Gao et al. [19], the Q-PRIMARY score might improve accuracy for detection of csPCa perhaps with addition of the “red flags” proposed in the current study. This might aid in reducing interobserver variability for separating PRIMARY-2 from PRIMARY-3 as previously discussed but needs to be further studied in larger cohorts.

We evaluated [^18^F]PSMA-1007 PET/MRI for second line biopsy guidance in a clinical setting after negative MRI or non-csPCa biopsies from PI-RADS 3–5 lesions, supporting the hypothesis that PRIMARY score is applicable regardless of PSMA-ligand. In general, we examined patients with high PSAd and found csPCa in 23.3% of patients and in 19.9% of lesions. We found csPCa in 18.2% of patients with PSAd between 0.15 and 0.20 ng/mL/cm³ and in 25.7% of patients with PSAd ≥ 0.20 ng/mL/cm³ but we found no csPCa below PSAd of 0.17 ng/mL/cm³. No method is perfect, which the case of both MRI- (PI-RADS-2) and PSMA-negative (PRIMARY-1/2) csPCa illustrates (Figs. 6 and 7). Even though false-positive PSMA PET lesions end-up being biopsied, the total number of biopsies per patient is substantially reduced (more than 50%) with the targeted approach [29].

Our data supports that the PRIMARY score can be used regardless of PSMA-ligand. Therefore, an approach with PSMA-guided biopsies should be feasible in all hospitals with access to PSMA PET/MRI or PET/CT, preferably using MRI/ultrasound fusion equipment, but alternatively with cognitive fusion biopsies. A limitation of the present study is that PRIMARY 1–2 lesions were not systematically biopsied, leading to very limited data on these. However, we find the results very encouraging, suggesting that the PRIMARY score can be safely applied with [¹⁸F]PSMA-1007 PET/MRI and may serve as second-line biopsy guidance tool in men with persistent suspicion of csPCa following MRI-first without suspicious areas on MRI or non-csPCa biopsies from PI-RADS 3–5 lesions. Recently, we initiated a prospective clinical trial to study the clinical impact in that setting (clinicaltrials.gov; NCT07258056).

## Conclusion

We prospectively evaluated the PRIMARY score for [¹⁸F]PSMA-1007, we identified benign features and potential “red flags” for PRIMARY-3 lesions. In patients with negative MRI exams or non-csPCa biopsies from PI-RADS 3–5 lesions but ongoing suspicion of csPCa, almost one in four harbored clinically significant disease. Interestingly, csPCa rates were substantial in both patients with PSAd between 0.15 and 0.20 ng/mL/cm³ and those above 0.20 ng/mL/cm³. While these findings support the utility of PSMA PET in guiding biopsy, no method is infallible, as illustrated by a case of multiple microfocal tumor infiltrations of csPCa that was negative on both PSMA PET and MRI.

## Supplementary Information

Supplementary Material 1.

## Data Availability

The data supporting the conclusions of the present paper is included.
